# Editorial: Community series in the role of angiogenesis and immune response in tumor microenvironment of solid tumor: volume II

**DOI:** 10.3389/fimmu.2023.1332519

**Published:** 2023-11-22

**Authors:** Haosheng Li, Zheyu Yang, Xin Lu, Ren Zhao, Xi Cheng

**Affiliations:** ^1^ Department of General Surgery, Ruijin Hospital, Shanghai Jiao Tong University School of Medicine, Shanghai, China; ^2^ Shanghai Institute of Digestive Surgery, Ruijin Hospital, Shanghai Jiao Tong University School of Medicine, Shanghai, China; ^3^ Department of Biological Sciences, University of Notre Dame, Notre Dame, IN, United States

**Keywords:** tumor microenvironment, angiogenesis, immune response, solid tumor, single cell sequence (scRNA-seq)

## Introduction

As continually updated in “The Hallmarks of Cancer” by Hanahan and Weinberg, the tumor microenvironment (TME) is now widely recognized to play an indispensable role in tumorigenesis and malignant progression (1–3). This heterogeneous and interacting collective consists of cancer cells, cancer stem cells, and various recruited stromal cell types which include vascular cells, fibroblasts, diverse immune cells (lymphocytes, monocytes/macrophages, and inflammatory cells), and extracellular matrix (ECM) (4). Half a century ago, Folkman proposed that solid tumors rely on the formation of new blood vessels from the pre-existing vasculature within their tumor environment to provide adequate nutrients and oxygen supply, making anti-angiogenic therapy beneficial for cancer treatment (5). Subsequently, extensive research has identified anti-angiogenic therapy as a cornerstone of modern cancer therapy (6). Furthermore, research has continually revealed that tumor endothelial cells exhibit heterogeneity and plasticity, and angiogenic factors are closely associated with the inflammatory response during tumor development (7, 8). This complexity in tumor vasculature functionality implies a greater diversity of cell-cell interactions within the TME than initially expected (9). Regulatory immune cells also secrete various cytokines and pro-angiogenic factors, accelerating tumor progression (10). The advent of single-cell sequencing (scRNA-seq) provides a tool for deciphering the tumor immune microenvironment. Compared to traditional methods, scRNA-seq can be employed to identify novel cell types and corresponding cellular states, deepening our understanding of the TME (11).

This special topic research, titled “Community Series in the Role of Angiogenesis and Immune Response in Tumor Microenvironment of Solid Tumor: Volume II”, comprises 10 original research articles, 4 review articles, 1 case report, 1 method paper, and 1 perspective piece, totaling 17 original contributions. These articles elucidate the latest advances in tumor angiogenesis, the molecular mechanisms of tumor-infiltrating lymphocytes (TILs) in different malignancies, the functions and roles of other immune cells such as NK cells and regulatory T cells (Tregs), and the applications of scRNA-seq techniques. In this editorial, we will discuss these aspects, and aim to provide new insights into anti-tumor angiogenesis therapy, alterations in the immune microenvironment, as well as the regulation of immune responses.

## Combining anti-angiogenic and immunotherapeutic approaches

Anti-angiogenic therapy stands as a viable tool for restoring immune cell infiltration within solid tumors, with combined therapies achieving greater success in immune-excluded and immune-desert tumors (12). Hu et al. provide an overview of the synergistic effects when anti-angiogenic agents are combined with immunotherapy in solid tumors, leading to improved drug resistance and cooperative inhibition of tumor growth and progression. Notably, this effect is observed in non-small-cell lung cancer, hepatocellular carcinoma, and renal cell carcinoma, as opposed to breast cancer, glioblastoma, and pancreatic ductal adenocarcinoma. To address efficacy concerns, the identification of sensitive biomarkers and the determination of appropriate combination dosages are imperative. These actions aim to augment immune cell infiltration and, consequently, enhance the effectiveness of immunotherapy. Shamshiripour et al. delve into the molecular mechanisms of abnormal angiogenesis in glioblastoma, discussing the applications and limitations of monoclonal antibodies, tyrosine kinase inhibitors, and aptamers in anti-angiogenic immunotherapy. Moreover, utilizing nanoparticles to deliver small interfering RNA (siRNA) across the blood-brain barrier represents a promising approach for the next generation of anti-angiogenic therapy, particularly in targeted brain delivery. Zhang et al. have identified a novel prognostic signature comprising four angiogenesis-related genes, AAG-related long non-coding RNAs (lncRNAs), including AC093278.2, NNT-AS1, CYTOR, and NUP50-DT, which serve as potential prognostic factors in clear cell renal cell carcinoma (KIRC). These lncRNAs show promise as independent prognostic indicators for KIRC patients. In a case report by Li et al., they document the case of a metastatic HCC patient who experienced recurrence post-surgery, and subsequently received a combination therapy of anti-angiogenic treatment and immune checkpoint inhibitors (lenvatinib and toripalimab). After seven months of treatment, the patient achieved complete remission, a status maintained until the paper’s submission, with a final calculation of progression-free survival at 24 months. The synergistic effects of anti-angiogenic therapy, intratumoral cryoablation, and immunotherapy have yielded highly favorable outcomes for the patient, although the precise mechanisms behind this synergistic treatment approach remain to be elucidated.

## Function and role of TILs

Immunocytes constitute a pivotal component within the intricate TME (13). The types and densities of TILs hold significant relevance for cancer progression and immunotherapeutic responses (14). Xiao et al. introduced the Tumor-Infiltration Immune Cell Proportion Estimator (TICPE) to estimate the proportions of immune cells in colorectal cancer and melanoma. Performance evaluations, which employed mRNA mixture expression data, scRNA-Seq data, immunohistochemistry data, and simulated bulk RNA-Seq samples, demonstrated its markedly superior accuracy compared to other methods. Cheng et al. observed that TILs and tertiary lymphoid structures (TLS) independently serve as prognostic factors in EBV-negative gastric cancer (EBVnGC), offering auxiliary indicators for gastric cancer prognosis. They established a nomogram model combining TILs grade and TLS status with other established prognostic factors, exhibiting good performance in calibration and external validation. Penny et al. identified 120 HLA-I phosphopeptides from primary CRC tumors, CRC liver metastases, and CRC cell lines using mass spectrometry. They evaluated the immune capacity of these post-translationally modified tumor antigens within tumors. PTM tumor antigens, namely HLA-I phosphopeptides, emerged as potential optimal targets for future immunotherapies, as they are targets for tumor-resident CD8 T cells. Wang et al. discovered a significant correlation between serum SDF-1 expression and TIL abundance in triple-negative breast cancer (TNBC) patients who underwent neoadjuvant chemotherapy (NAC) following standard radical surgery. SDF-1, when considered in conjunction with TILs, aids in identifying patients who would benefit from chemotherapy, thereby enhancing the pathological complete response (pCR) rate and preventing disease recurrence in non-pCR patients. Regarding TIL-based cancer therapy, Aydin et al. underscore the indispensability of identifying patients with bladder cancer (BC) who generate the optimal quantity of active TILs. Tumors in both primary and lymph node metastases in BC patients can produce tumor-specific TIL responses, justifying clinical trials to validate TILs as a rational treatment strategy for BC patients. Elkoshi proposes an explanation for the inverse correlation between tumor-infiltrating Tregs and survival in various cancer types. The frequency or proportion of Tregs and CD8+ T cells at the tumor site in the TME is mutually correlated. Consequently, this ratio exhibits less variation in frequency compared to both lymphocyte populations separately. However, if one of these lymphocyte populations experiences substantial frequency fluctuations, opting for the lymphocyte population with lower frequency variation can enhance survival rates, particularly when the intra-tumor frequencies of the two lymphocyte types are inversely related. Selecting such optimal prognostic markers in this manner may also serve as the best predictive factor for cancer checkpoint inhibitor therapies.

## ScRNA-seq reveals insights into the TME

ScRNA-seq technology enables a more comprehensive analysis of genetic and protein information differences between cells, allowing for the acquisition of individual cell genomic sequence information and a deeper investigation into the cellular characteristics and interactions within the TME (15). Wen et al. summarized the heterogeneity of the TME in colorectal cancer, highlighting the individualized and highly mutated nature of tumor epithelial cells in each patient. Various immune cells and inflammatory chemokines within the TME interact and influence one another, promoting tumor progression and thereby impacting tumor recurrence and treatment response. He et al. focused on the applications of scRNA-seq in revealing heterogeneity, microenvironment characteristics, and drug resistance in retinoblastoma (RB) and uveal melanoma (UM). This approach holds promise for identifying new biomarkers for diagnosis and targeted therapy. Ziblat et al. conducted phenotypic analysis of peripheral blood NK cells (PBNK) and tumor-infiltrating NK cells (TINK) from clear cell renal cell carcinoma (ccRCC) patients. PBNK in ccRCC patients exhibited an activated phenotype marked by the expression of CD25, CD69, and CD62L, while TINK showed reduced expression of DNAM-1, NKp30, NKp46, NKp80, and CD16, suggesting a more suppressive phenotype. Jiang et al. discovered that Galectin A-Related Protein (GARP) maintains Treg-mediated immune tolerance in gastric cancer. Upregulation of GARP was associated with increased FOXP3+ Treg and CD4+ T cell infiltration and positively correlated with CTLA-4 and PD-L1 expression. Furthermore, the role of CD4+ T cell immune signaling in premalignant gastric cancer may hold clinical significance, offering new insights into immune therapy approaches.

## Emerging avenues in immunotherapy

Novel and advancing approaches in the field of immunotherapy are continually expanding, offering innovative avenues for the treatment of cancer (16). These emerging strategies encompass a diverse array of immunomodulatory techniques and novel therapeutic targets, promising the potential to enhance the effectiveness and precision of immunotherapeutic interventions (17). Zhang et al. identified the optimal single-chain variable fragment and investigated its biological functionality to further enhance the therapeutic potential of CAR-T cells targeting CEA-positive cancers. Proper affinity can improve the functionality of CAR-T cells based on different CAR-T types. Four CEA-targeting CAR-T cell sources were screened and compared, with M5A CAR-T cells demonstrating stable CAR expression, moderate affinity, cytokine secretion, and excellent anti-tumor capabilities both *in vitro* and *in vivo*. Xu et al. identified three distinct forms of programmed cell death in colorectal cancer, each associated with specific TME cell infiltration characteristics related to immune exclusion, immune desert, and immune-inflamed phenotypes. Patients with higher COPsig scores exhibited longer overall survival, lower immune cell and stromal infiltration, and a greater tumor mutational burden. Pan et al. discovered significant upregulation of soluble immune checkpoint-related proteins, BTLA, CD28, CD137, GITR, and LAG-3, in pancreatic ductal adenocarcinoma that were significantly associated with prognosis. Patients classified as having a soluble immune low subtype based on these biomarkers exhibited superior overall survival compared to those classified as having a soluble immune high subtype.

## Summary and prospective

This topic emphasizes the multifaceted dynamics of the TME, underscoring the pivotal roles of angiogenesis and immune regulation in cancer therapy. By integrating these two distinct yet interconnected processes, the aim is to enhance our comprehension of cancer treatment modalities. Furthermore, we delve into the functional significance of TILs from a cancer biology perspective. The emergence of avenues such as scRNA-seq, CAR-T therapy, programmed cell death, soluble immune checkpoints, and others has opened new paths for deciphering the communication occurring within the TME and developing innovative approaches for therapy. We aspire to advance our understanding of the roles of these elements in cancer immunity and therapeutic strategies, thereby contributing to the development of novel approaches for cancer treatment.

In conclusion, the articles included in this topic provide a new direction for the development of angiogenesis and immune response in the TME. We would also like to express our sincere gratitude to all authors, reviewers, and the editorial team of *Frontiers in Immunology* for their devotion and assistance in the process of reviewing and publishing all these studies in this Research Topic. Simultaneously, we believe that with the tireless efforts of researchers worldwide, effective immune checkpoint targets like PD-1 and PD-L1 will continue to be discovered, offering new hope for cancer patients globally. The relentless exploration at the molecular level, facilitated by novel tools, holds the promise of a future where cancer can be effectively treated.

## Author contributions

HL: Writing – original draft, Writing – review & editing. ZY: Writing – review & editing. XL: Writing – review & editing. RZ: Writing – review & editing. XC: Writing – review & editing.
